# Identification of the CYP19A1-GPER1 axis as a critical oncogenic driver in hepatocellular carcinoma via AKT activation

**DOI:** 10.1186/s12967-026-08246-3

**Published:** 2026-05-19

**Authors:** Hang Zhai, Jicai Wang, Yongfei He, Shengjie Hong, Kai Huang, Shuai Hu, Shuang Hao, Guangquan Zhang, Xianjie Shi

**Affiliations:** 1 Department of Hepatobiliary and Pancreatic Surgery, The Eighth Affiliated Hospital, Sun Yat-Sen University, Shenzhen, 518033 China; 2 Department of Hepatobiliary Surgery, The First Affiliated Hospital of Guangxi Medical University, Shuang-Yong Rd. 6, Nanning, 530021 Guangxi Zhuang Autonomous Region People’s Republic of China

**Keywords:** Lipid metabolism, CYP19A1, GPER1, HCC

## Abstract

**Background:**

Hepatocellular carcinoma (HCC) has limited therapeutic efficacy at advanced stages. Lipid metabolism reprogramming drives HCC progression; however, the functional crosstalk between CYP19A1, GPER1, and lipid metabolic dysregulation in HCC remains unclear.

**Methods:**

Integrated analyses of The Cancer Genome Atlas Liver Hepatocellular Carcinoma data were performed. Clinical validation, functional assays, rescue experiments, in vivo xenografts, and molecular docking were conducted.

**Results:**

CYP19A1 was identified as a prognostic hub in HCC, with overexpression correlating with advanced T/BCLC stages (*p* < 0.05) and poor survival (log-rank *p* < 0.05). Gene set enrichment analysis linked CYP19A1 to dysregulated lipid metabolism, pro-tumorigenic signaling, and immune infiltration. GPER1 was found to be a critical effector of CYP19A1, mediating its effects on proliferation, migration, invasion, epithelial–mesenchymal transition, and protein kinase B activation. CYP19A1 knockdown reduced tumor burden in vivo, whereas *GPER1* overexpression rescued this phenotype.

**Conclusion:**

The CYP19A1-GPER1 axis represents a critical oncogenic driver in HCC, providing a mechanistic insight to guide future therapeutic development.

**Supplementary Information:**

The online version contains supplementary material available at 10.1186/s12967-026-08246-3.

## Introduction

Cancer remains one of the leading causes of death worldwide, posing a major public health challenge [1–3]. Liver cancer is a highly prevalent and lethal malignancy, with 865,000 new cases and 758,000 deaths worldwide in 2022, ranking sixth in incidence and third in mortality [4]. Primary liver cancer includes intrahepatic cholangiocarcinoma (ICC) and hepatocellular carcinoma (HCC), with HCC accounting for approximately 75–85% of cases and ICC for 15–25% [4, 5]. Key causes of HCC include viral hepatitis (55–60% of cases), aflatoxin exposure, heavy alcohol use, obesity, type 2 diabetes, and smoking [4–7]. Advances in first-line systemic chemotherapy have improved survival and perioperative outcomes; however, significant challenges remain, including hepatic insufficiency, limited efficacy against extrahepatic metastasis, inadequate biomarkers, high recurrence rates, and inconsistent standards for conversion therapy, highlighting the urgent need for novel therapeutic strategies [5, 8–16].

Lipid metabolism reprogramming, including dysregulated synthesis, catabolism, and signaling of lipids, promotes tumor progression by fueling proliferation, metastasis, and stemness [17–25]. It modulates the tumor microenvironment and therapeutic resistance, emerging as a pivotal target across cancers [17, 18, 26–28]. Cytochrome P450 family 19 subfamily A member 1 (CYP19A1), a lipid metabolism-related protein essential for estrogen biosynthesis, exerts multifaceted oncogenic roles in diverse cancers [29–32]. G protein-coupled estrogen receptor 1 (GPER1, also named GPR30) exacerbates HCC progression by promoting hepatocyte proliferation, liver growth, tumorigenesis and development, and participating in the immunosuppressive microenvironment; meanwhile, GPER1 enhances the survival of HCC cells by inducing mitophagy, thereby contributing to therapeutic resistance [33, 34]. Upon estrogen stimulation, GPER1 regulates the stability and nuclear accumulation of SIN1, a key subunit of mTORC2, thereby enhancing mTORC2-mediated phosphorylation of AKT at the S473 site, and consequently promoting hepatocyte proliferation and HCC progression [33, 35]. In addition, estrogen-activated GPER1 regulates the phosphatidylinositol 3-kinase (PI3K)-AKT-mTORC1 signaling axis, driving hepatocyte cycle progression and liver growth. This mechanism has been confirmed in zebrafish models and human hepatocytes [34]. Furthermore, cepharanthine hydrochloride targets GPER1 to induce mitophagy in HCC cells, thereby affecting cell survival, highlighting the role of GPER1 in HCC cellular processes [35]. However, the functional crosstalk between CYP19A1 and GPER1 in HCC remains incompletely understood. Given that CYP19A1 is the key enzyme catalyzing estrogen biosynthesis and GPER1 functions as an estrogen receptor [36, 37], we hypothesized that CYP19A1-derived estrogens may serve as ligands to activate GPER1 signaling in an autocrine or paracrine manner, thereby promoting HCC progression.

To fill this gap, we integrated the analysis of The Cancer Genome Atlas data with protein interaction networks and survival modeling, identifying *CYP19A1* as a central prognostic hub with its overexpression correlating with poor clinical outcomes. Clinical validation identified CYP19A1 as an independent prognostic marker associated with advanced-stage HCC and reduced OS. Rescue and in vivo studies confirmed that GPER1 is essential for CYP19A1-driven phenotypes, establishing the CYP19A1-GPER1 axis as a therapeutically targetable node linking metabolic dysregulation, immune evasion, and metastasis.

## Methods

### Data acquisition

The transcriptomic and clinical information were obtained from The Cancer Genome Atlas Liver Hepatocellular Carcinoma (TCGA-LIHC) cohort. A predefined list of lipid metabolism-related genes (LMGs) was obtained from Reactome (https://reactome.org/download-data/).

### Functional enrichment and protein-protein interaction (PPI) analysis

Enrichment analyses for gene ontology (GO) biological processes and Kyoto encyclopedia of genes and genomes (KEGG) pathways were conducted on differentially expressed LMGs through the clusterProfiler R package [38–40]. Gene set enrichment analysis (GSEA) was performed on pre-ranked gene lists based on correlation with CYP19A1 expression. Hallmark gene sets (MSigDB) were analyzed. PPI among the differentially expressed LMGs was predicted via the STRING database.

### Immune infiltration analysis

The CIBERSORTx algorithm was employed to quantify immune-cell infiltration in RNA-sequencing data from the TCGA-LIHC cohort, and assessed the association between CYP19A1 expression levels and these infiltration profiles using Spearman’s rank correlation analysis.

### Molecular docking

Human CYP19A1 was retrieved from UniProt and prepared via bond order assignment, hydrogen addition, removal of water/cofactors, hydrogen bond network optimization, and OPLS_4 force field energy minimization. Sorafenib, lenvatinib, and regorafenib from PubChem were prepared via Schrödinger Ligprep/Epik (OPLS_4, stereoisomers/protonation states). CYP19A1 (3S79) was prepared (Protein Preparation Wizard), and molecular docking was performed using Schrödinger Glide in standard precision mode with a ligand-defined binding pocket. Up to 10 poses per ligand were retained following post-docking minimization, and top-scoring conformations were analyzed. GlideScore < −5 indicative of potential binding, <−6 indicative of favorable binding.

### Patient samples and cell lines

Paired HCC tumor and adjacent non-tumorous liver tissue samples were acquired from The Eighth Affiliated Hospital of Sun Yat-sen University, upon obtaining written informed consent and approval from the Institutional Review Board (2024-098-01). HCC cell lines (HCCLM3, Huh7, Hep3B, and HepG2) were procured from Procell (Wuhan, China); MHCC97L and MHCC97H were obtained from Servicebio (Wuhan, China). All cell lines were authenticated by short tandem repeat profiling. All cells were incubated in a humidified atmosphere with 5% CO_2_ at 37 °C, with specific culture media as follows: Hep3B and HepG2 in MEM contained non-essential amino acids, 1% penicillin-streptomycin (P/S) and 10% fetal bovine serum (FBS); HCCLM3 in DMEM with 1% P/S and 20% FBS; MHCC97H, MHCC97L, and Huh7 in DMEM supplemented with 10% FBS and 1% P/S.

### Quantitative real-time PCR (qPCR) assay

The Evo M-MLV RT Mix Kit (AG11728) was used for cDNA synthesis: a two-step protocol (gDNA removal, RT at 37 °C/15 min, and inactivation at 85 °C) for samples with high gDNA and an all-in-one method for high-quality RNA. NRT controls were included. qPCR was performed using the SYBR Green Premix Kit (AG11701) on the ABI QuantStudio™5. 20 µL system: 10 µL premix, ≤100 ng cDNA, 0.4 µL each primer (10 μM), 0.4 µL ROX (4 μM), RNase-free water. The standard curve had 99.0%–99.7% efficiency, R^2^ ≥ 0.998. The 2^–ΔΔCt^ method was used for the calculation of relative expression. The primers used in this study are provided in Supplementary Table S1.

### Establishment of stable cell lines

Stable cell lines were generated through lentiviral transduction and antibiotic selection. CYP19A1 was overexpressed in Hep3B cells and knocked down in HepG2 cells using pLV3-based vectors (Puromycin selection). Rescue experiments were performed by overexpressing GPER1 in HepG2 cells and silencing GPER1 in Hep3B cells using a Neomycin-resistant vector (G418 selection). Knockdown and overexpression efficiencies were confirmed by qPCR and Western blotting (Supplementary Fig. S1). The full -length open reading frame sequences of CYP19A1 and GPER1, and shRNA oligonucleotide sequences are provided in Supplementary Tables S2 and S3.

### Immunohistochemistry (IHC)

Freshly dissected tissues were fixed (>24 h), trimmed, dehydrated through graded alcohols, and paraffin-embedded at 65 °C. Sections (4 µm) were cut, flattened at 40 °C, mounted onto slides, and baked at 60 °C. For IHC: sections were deparaffinized and rehydrated; antigen retrieval was performed using citrate buffer (pH 6.0) or EDTA buffer (pH 9.0) according to the manufacturer’s instructions, followed by phosphate buffer saline (PBS) washes. Endogenous peroxidase activity was blocked with 3% H_2_O_2_ for 25 min in the dark, followed by PBS washes. Sections were blocked with 3% BSA for 30 min at room temperature, and incubated with primary antibodies at 4 °C overnight. The following primary antibodies were used: anti-CYP19A1 (ABclonal, A12238, 1:800), anti-GPER1 (ABclonal, A10217, 1:200), anti-E-cadherin (ABclonal, A20798, 1:400), anti-Vimentin (ABclonal, A19607, 1:1000), and anti-Ki67 (ABclonal, A20018, 1:200). After PBS washes, sections were incubated with biotin-labeled goat anti-rabbit IgG (ready-to-use; from the immunohistochemistry kit, Bioss, IHC001) for 50 min at room temperature, followed by PBS washes. Streptavidin-HRP (ready-to-use, from the same kit) was applied according to the manufacturer’s instructions. DAB chromogenesis was performed under microscopic monitoring and terminated with tap water. Hematoxylin counterstaining was performed for 3 min, followed by differentiation, bluing, and dehydration (75% alcohol to xylene I, 5 min each), and sections were mounted. Results were observed under bright-field microscopy. Target protein expression was quantified by average optical density (AOD = IOD/area), and Ki-67-positive cells were counted manually.

### Western blotting

Samples were homogenized in lysis buffer on ice, followed by centrifugation at 14,000 rpm for 15 min at 4 °C. Supernatants were pipetted into pre-chilled microtubes; aliquots were used for protein quantification. Lysates in loading buffer were heated to 100 °C for 10 min. Proteins were loaded onto SDS-polyacrylamide gels. Membranes were blocked with 5% non-fat milk in TBST for 1 h at room temperature, and incubated with primary antibodies at 4 °C overnight. The following primary antibodies were used: anti-CYP19A1 (ABclonal, A12238, 1:1000), anti-GPER1 (ABclonal, A10217, 1:1000), anti-E-cadherin (ABclonal, A20798, 1:1000), anti-N-cadherin (ABclonal, A19083, 1:1000), anti-vimentin (ABclonal, A19607, 1:10000), anti-phospho-mTOR (Proteintech, 80596-1-RR, 1:1000), anti-mTOR (Proteintech, 81670-1-RR, 1:1000), anti-phospho-p70(S6K) (Proteintech, 82373-1-RR, 1:1000), anti-p70(S6K) (Proteintech, 66638-1-Ig, 1:1000), anti-GSK3B (Proteintech, 82061-1-RR, 1:1000), anti-phospho-GSK3B (Proteintech, 67558-1-Ig, 1:1000), anti-phospho-PDPK1 (Proteintech, 85223-3-RR, 1:1000), anti-PDPK1 (Proteintech, 17086-1-AP, 1:1000), anti-phospho-PDPK1 (Proteintech, 85223-3-RR, 1:1000), anti-AKT (ABclonal, A18675, 1:1000), anti-phospho-AKT (ABclonal, AP1259, 1:1000), anti-vinculin (ABclonal, A2752, 1:100000), and anti-β-actin (Proteintech, 81115-1-RR, 1:10000). After washing six times with TBST (5 min each), membranes were incubated with HRP-conjugated secondary antibodies for 1 h at room temperature. The secondary antibodies used were goat anti-rabbit IgG (Proteintech, SA00001-2, 1:10000). Chemiluminescent signals were detected using an enhanced chemiluminescence substrate and imaged via darkroom development.

### ELISA

HCC cells were seeded in 6-well plates and cultured in phenol red-free medium supplemented with 10% charcoal-stripped FBS for 48 h for estrogen production assays. Conditioned media were collected, and 17β-estradiol (E2) concentrations were measured using a human 17β-Estradiol ELISA kit (CUSABIO, CSB-E05108h) according to the manufacturer’s instructions. Absorbance was measured at 450 nm using a microplate reader, and E2 concentrations were calculated from a standard curve. Each experiment was performed in triplicate.

### Colony formation assay

Cells with high viability were dissociated. After centrifugation, the cells were resuspended in a single-cell suspension using gentle pipetting. A 6-well plate was employed as the cloning culture plate, and cells were plated at 1000 cells/well. Post-seeding, the plate was swirled gently to achieve uniform cell distribution. Following approximately 2 weeks of incubation, cells were fixed with 4% paraformaldehyde and stained with 0.5% crystal violet, and imaged.

### EdU assay

A 96-well plate was employed as the cloning culture plate, and cells were plated at 1000 cells/well. After overnight culture, pre-warmed (37 ℃) 10 µM EdU working solution (from BeyoClick™ EdU Cell Proliferation Kit with Alexa Fluor 488, Cat#C0071S, Beyotime) was added for 2 h incubation. Cells were subjected to fixation with 4% paraformaldehyde (15 min, RT), washed 3 × (3–5 min), permeabilized (10–15 min, RT), and incubated with Click reaction solution. After 3 washes, 1X Hoechst 33342 was added, followed by 3 washes before fluorescence microscopy observation.

### Transwell assay

For migration assay: Transwell inserts were pre-incubated with serum-free medium for 30 min. Serum-free cell suspensions and serum-containing medium were added to the upper and lower chambers, respectively. For invasion assay: Matrigel (thawed at 4 ℃) was diluted to 1 mg/mL with serum-free medium using pre-chilled tips, 60 µL added to inserts, incubated at 37 °C for 1–3 h, and hydrated for 30 min. Lower chambers contained 500 µL 10% FBS medium, while upper chambers received serum-free cell suspensions. After 48 h incubation, cells that did not migrate were wiped off, fixed with 4% paraformaldehyde, stained with 0.1% crystal violet, and the migrated cells counted microscopically.

### Wound healing assay

Cells in the log phase were subjected to trypsinization to form single-cell suspensions, plated in 6-well plates, and incubated at 37 °C with 5% CO_2_ until reaching 95% confluence. Scratches were made using a 10 μL pipette tip. Scratches were created with a 10 µL micropipette tip. Cells were washed to remove detached cells and incubated in serum-free medium. Images were acquired at 0 and 24 h using a microscope. The mean scratch area was calculated using ImageJ software.

### In vivo xenograft studies

In vivo experiments were performed using 4-week-old male BALB/c nude mice. Orthotopic and subcutaneous liver tumor models were developed using subcutaneous or orthotopic liver injection of HepG2 cells, transduced with shNC, shCYP19A1, shCYP19A1 with empty vector, or shCYP19A1 with vector expressing GPER1, respectively. Each group consisted of five mice. Mice were euthanized four weeks after injection, and tumors were harvested for analysis.

### Statistical analysis

All statistical analyses were conducted using R or GraphPad Prism software. Data are expressed as mean ± standard deviation (SD). Differences between two groups were assessed using Student’s t-test (parametric) or Mann–Whitney U test (non-parametric). Differences among multiple groups were analyzed using one-way analysis of variance with Tukey’s post-hoc or Kruskal–Wallis test with Dunn’s post-hoc test. Correlation analyses used Pearson’s or Spearman’s correlation coefficients. Survival differences were assessed using the log-rank test. *p* values < 0.05 were considered statistically significant (*p < 0.05, **p < 0.01, ***p < 0.001).

## Results

### Prognostic lipid metabolism genes in HCC identified via integrated analysis

**Fig. 1:**
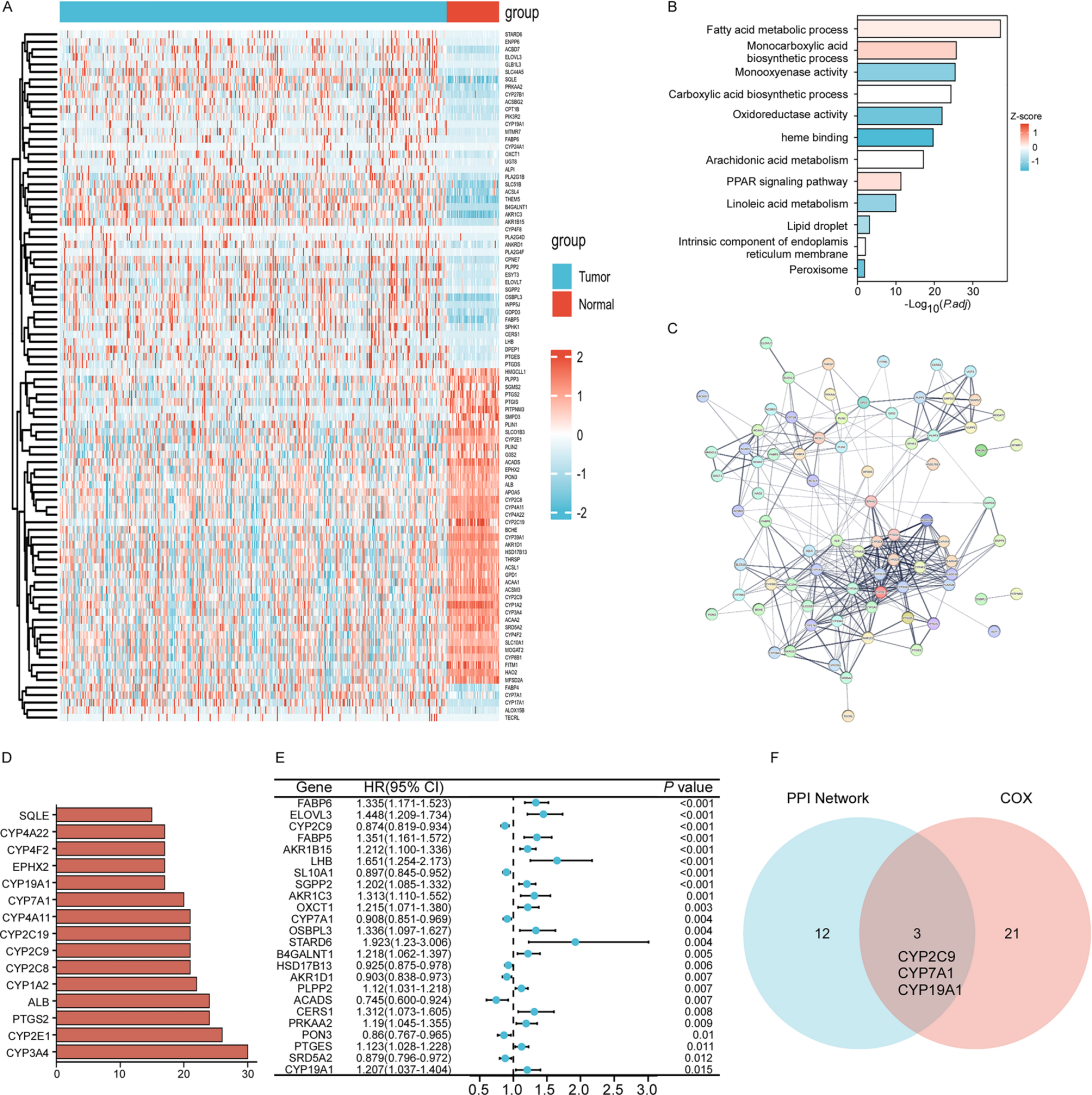
Identification of prognostic LMGs in HCC via integrated analysis. (**A**) Heatmap displaying differentially expressed LMGs between HCC tumor tissues and adjacent normal liver tissues in LIHC cohort. (**B**) Enrichment analysis of differentially expressed LMGs. (**C**) PPI network of differentially expressed LMGs. (**D**) Bar chart ranking the top 15 hub genes. (**E**) Forest plot of univariate COX proportional hazards analysis, demonstrating LMGs significantly correlated with OS in LIHC. (**F**) Venn diagram depicting the intersection of LMGs from the PPI network and those associated with OS in the COX analysis

Analysis of gene expression differences was conducted between HCC tumor specimens and paired peritumoral normal specimens using data from TCGA-LIHC (Fig. 1A). Enrichment analysis of these differentially expressed LMGs identified significant associations with key biological pathways, including the following: monooxygenase activity, oxidoreductase activity, heme binding, and other related pathways (Fig. 1B). To explore potential mechanisms, a PPI network was built with the STRING database, visualizing interrelationships among differentially expressed genes associated with lipid metabolism (Fig. 1C). A bar chart ranked the top 15 hub genes based on their number of nodes within the network (Fig. 1D). Univariate Cyclooxugenase (COX) regression analysis demonstrated that some LMGs were associated with the HCC prognosis (Fig. 1E). A Venn diagram was used to highlight the overlap between LMGs detected in PPI network analysis and those significantly correlated with OS in the univariate Cox analysis. This intersection revealed three key genes: *CYP2C9*, *CYP7A1*, and *CYP19A1* (Fig. 1F).

### Elevated CYP19A1 dysregulates metabolism, modulates immunity, and binds HCC therapeutics

**Fig. 2:**
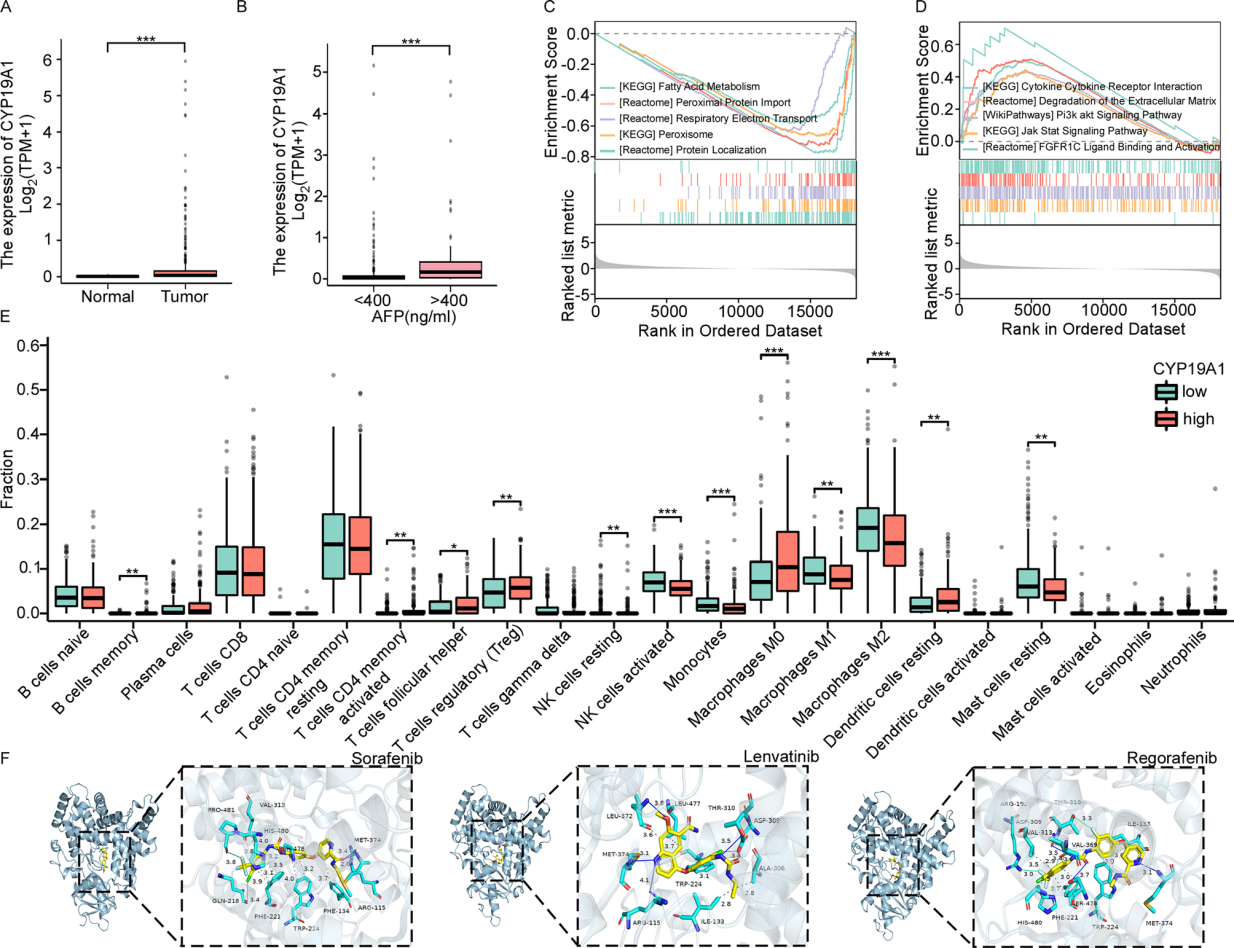
CYP19A1 overexpression in HCC dysregulates metabolism, modulates immunity, and interacts with therapeutic agents. (**A**) Box plot illustrates CYP19A1 mRNA expression levels in HCC tumor and adjacent normal tissues (Mann–Whitney U test). (**B**) The box plot presents the relationship between CYP19A1 mRNA expression levels and AFP levels (Mann–Whitney U test). (**C**, **D**) GSEA plots illustrating pathways negatively (**C**) and positively (**D**) correlated with CYP19A1 expression. (**E**) Correlation of CYP19A1 expression with the relative abundance of 22 immune cell types (Wilcoxon rank-sum test). (**F**) Docking conformations and interaction force analysis of CYP19A1 with sorafenib, lenvatinib, and regorafenib. Blue sticks: amino acid residues; yellow: small molecules; blue solid lines: hydrogen bonds; green solid lines: halogen bonds; gray dashed lines: hydrophobic interactions. Data are presented as mean ± SD. *p < 0.05, **p < 0.01, ***p < 0.001

CYP19A1 mRNA expression was significantly elevated in tumor tissues compared to adjacent normal liver tissues within the TCGA-LIHC database (Fig. 2A). Moreover, elevated mRNA expression of CYP19A1 was observed in tumor tissues with high AFP levels compared to those with low AFP levels in the TCGA-LIHC cohort (Fig. 2B). GSEA revealed that CYP19A1 expression exhibited negative correlations with pathways including fatty acid metabolism, peroxisomal protein import, respiratory electron transport, peroxisome, and protein localization (Fig. 2C). Conversely, CYP19A1 expression was positively correlated with pro-cancer signaling pathways such as PI3K-Akt and JAK-STAT, as well as other related pathways (Fig. 2D). Furthermore, CIBERSORT analysis demonstrated that CYP19A1 expression levels were significantly associated with the infiltrate levels of 12 distinct immune cell types (Fig. 2E). Given the clinical relevance of sorafenib, lenvatinib, and regorafenib as common therapeutic agents for HCC, molecular docking simulations were performed to assess their potential interaction with CYP19A1. The calculated docking scores were −5.034 for sorafenib, −6.228 for lenvatinib, and −6.288 for regorafenib (Fig. 2F). These findings suggest that CYP19A1, overexpressed in HCC, may promote tumor progression by dysregulating lipid metabolism, modulating immune cell infiltration, and interacting with pro-cancer pathways while also exhibiting potential implications for response to targeted therapies such as sorafenib, lenvatinib, and regorafenib.

### Validated CYP19A1 overexpression predicts advanced stage and poor survival in HCC

**Fig. 3:**
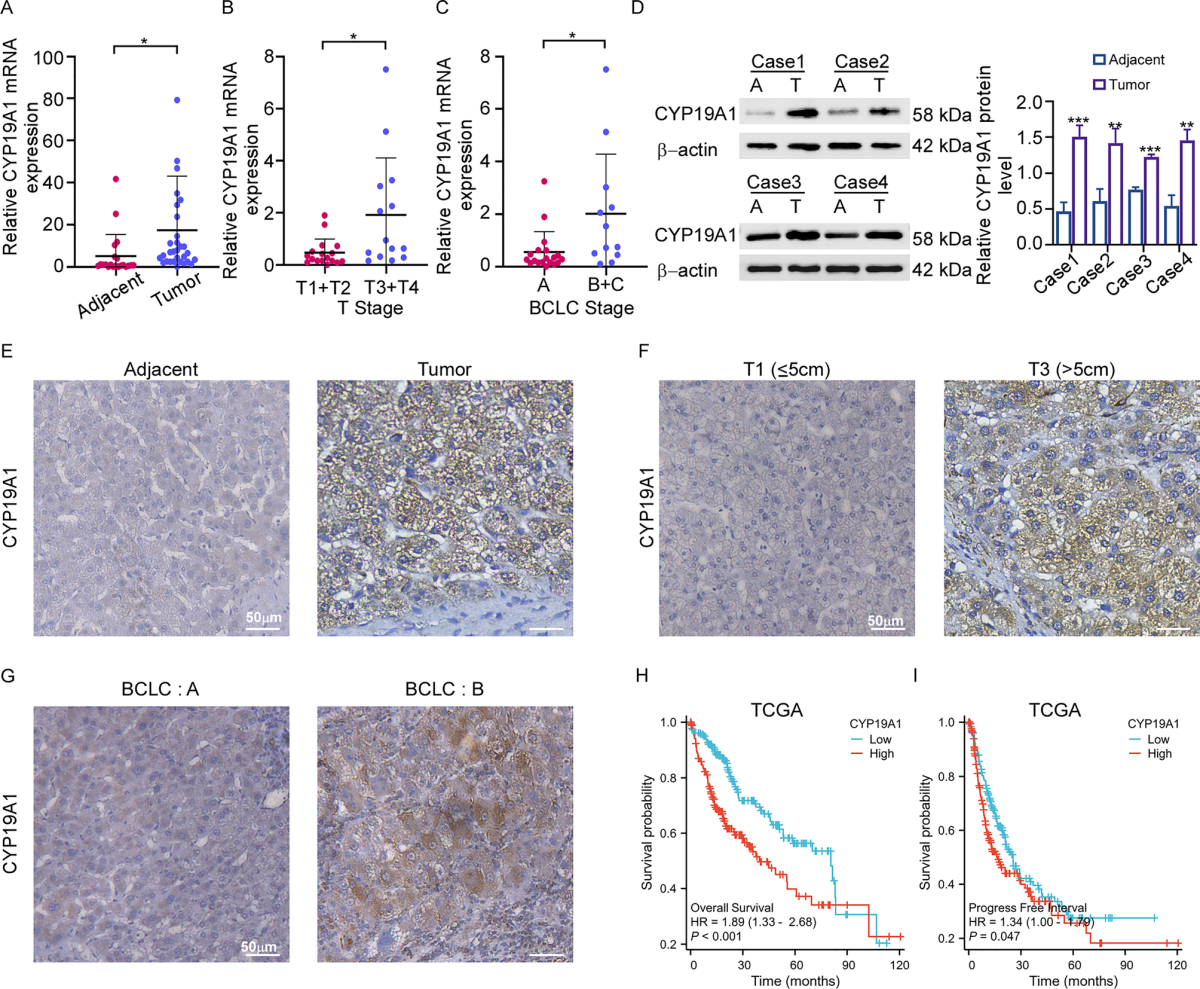
Clinical validation of CYP19A1 as a prognostic biomarker in HCC. (**A**) qPCR analysis demonstrates CYP19A1 mRNA expression in HCC tumors and adjacent normal tissues (Mann–Whitney U test). (**B**, **C**) qPCR results reveal CYP19A1 mRNA expression in T stages (**B**) and BCLC stages (**C**) (Mann–Whitney U test). (**D**) Western blotting analysis displays CYP19A1 protein expression in clinical specimens. Molecular weight markers and quantitative analysis of protein expression levels from three independent experiments are illustrated on the right (parametric or non-parametric tests are used as appropriate). (**E**) IHC of CYP19A1 in paired tumor and adjacent tissues. Scale bar: 50 μm. (**F**, **G**) IHC demonstrates CYP19A1 protein levels across T stages (**F**) and BCLC stages (**G**). Scale bar: 50 μm. (**H**, **I**) Kaplan–Meier plots for OS (**H**) and progression-free interval (**I**) grouped by CYP19A1 expression (log-rank test). Data are presented as mean ± SD. *p < 0.05, **p < 0.01, ***p < 0.001

Clinical validation in HCC patient samples confirmed significant CYP19A1 overexpression. qPCR and IHC analyses demonstrated markedly higher CYP19A1 mRNA and protein expression levels in tumor tissues compared to matched adjacent non-tumorous liver tissues (Fig. 3A and E). Furthermore, CYP19A1 expression exhibited a positive correlation with increasing T stage (Fig. 3B and F) and advanced BCLC stage (Fig. 3C and G). Western blotting further corroborated the increased CYP19A1 protein expression in tumor versus adjacent normal tissues (Fig. 3D). Consistently, analysis of the TCGA-LIHC database revealed that patients with high CYP19A1 expression exhibited significantly shorter overall survival (OS) (Fig. 3H) and progression-free interval (PFI) (Fig. 3I). These findings robustly establish CYP19A1 overexpression as a clinically validated biomarker in HCC that correlates with advancing disease stage (T and BCLC) and serves as a significant adverse prognostic factor for overall survival (OS) and PFI, highlighting its potential clinical relevance.

#### CYP19A1 promotes HCC cell proliferation, migration, invasion, and EMT via AKT activation

**Fig. 4:**
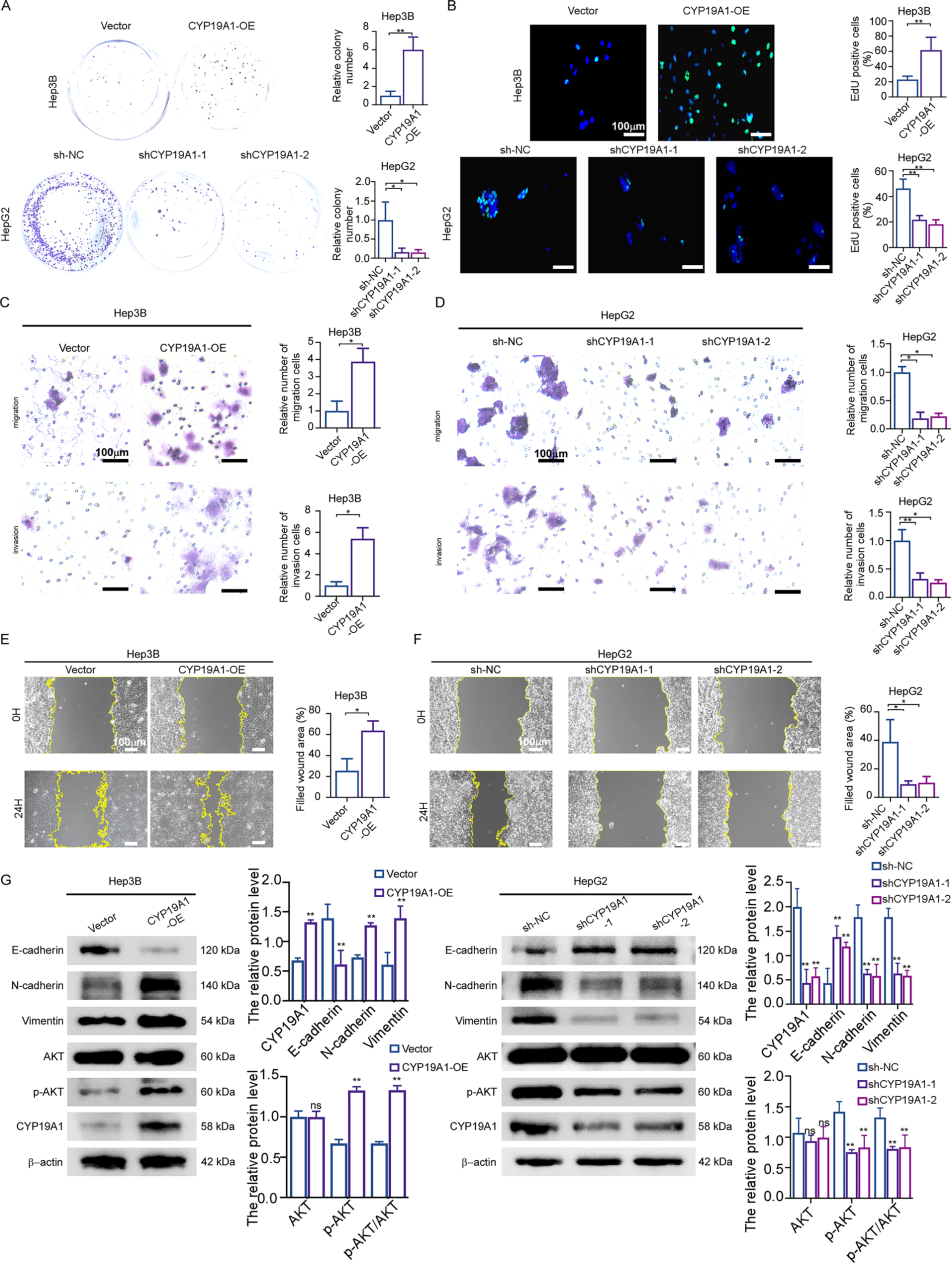
CYP19A1 modulates HCC cell proliferation, migration, invasion, and EMT through AKT signaling. (**A**, **B**) Cell proliferation is evaluated using colony formation assay (**A**) and EdU assay (**B**) in HCC cells. (**C**, **D**) Transwell assays are performed to assess cell invasion and migration in Hep3B (**C**) and HepG2 (**D**) cells. (**E**, **F**) Wound healing assays verify the effect of CYP19A1 overexpression or knockdown on cell migration ability in Hep3B (**E**) and HepG2 (**F**) cells. (**G**) Western blotting of EMT markers and AKT phosphorylation in Hep3B and HepG2 cells. Molecular weight markers and quantitative analysis of protein expression levels from three independent experiments are presented on the right. Parametric or non-parametric tests are used as appropriate based on data distribution. Scale bar: 100 μm. Data are presented as mean ± SD; **ns, not significant; *p < 0.05, **p < 0.01

To further verify the expression levels of CYP19A1 across HCC cell lines, qPCR analysis revealed that CYP19A1 mRNA expression was the lowest in Hep3B cells and the highest in HepG2 cells (Fig. S1A). Accordingly, *CYP19A1* was upregulated in Hep3B cells, while it was silenced in HepG2 cells (Fig. S1B–D). To confirm CYP19A1-mediated estrogen biosynthesis in HCC cells, 17β-estradiol (E2) levels in cell culture media were measured via human 17β-E2 ELISA. CYP19A1 overexpression significantly increased E2 secretion in Hep3B cells compared to the vector control (Fig. S2A). Conversely, CYP19A1 knockdown remarkably reduced E2 concentrations in HepG2 cell media relative to sh-NC (Fig. S2B). EdU and colony formation assays demonstrated that CYP19A1 overexpression in Hep3B cells enhanced HCC cell proliferation, whereas CYP19A1 knockdown in HepG2 cells reduced this ability (Fig. 4A and B). CYP19A1 overexpression significantly enhanced Hep3B cell viability after sorafenib treatment (Fig. S2C). Conversely, CYP19A1 depletion reduced HepG2 cell viability under sorafenib stimulation (Fig. S2D). Transwell assays revealed that CYP19A1 overexpression in Hep3B cells promoted HCC cell migration and invasion, while its knockdown in HepG2 cells diminished these abilities (Fig. 4C and D), and wound healing assays confirmed similar effects on migration, with enhanced motility in CYP19A1-overexpressing Hep3B cells and reduced migration in CYP19A1-knockdown HepG2 cells (Fig. 4E and F). Western blotting revealed that CYP19A1 overexpression in Hep3B cells altered EMT markers—decreasing E-cadherin and increasing N-cadherin and vimentin—along with enhanced AKT phosphorylation, whereas the opposite effects were observed upon CYP19A1 knockdown in HepG2 cells (Fig. 4G).

#### GPER1 exerts oncogenic effects in HCC by promoting malignant phenotypes and (epithelial-mesenchymal transition) EMT via AKT signaling activation

**Fig. 5:**
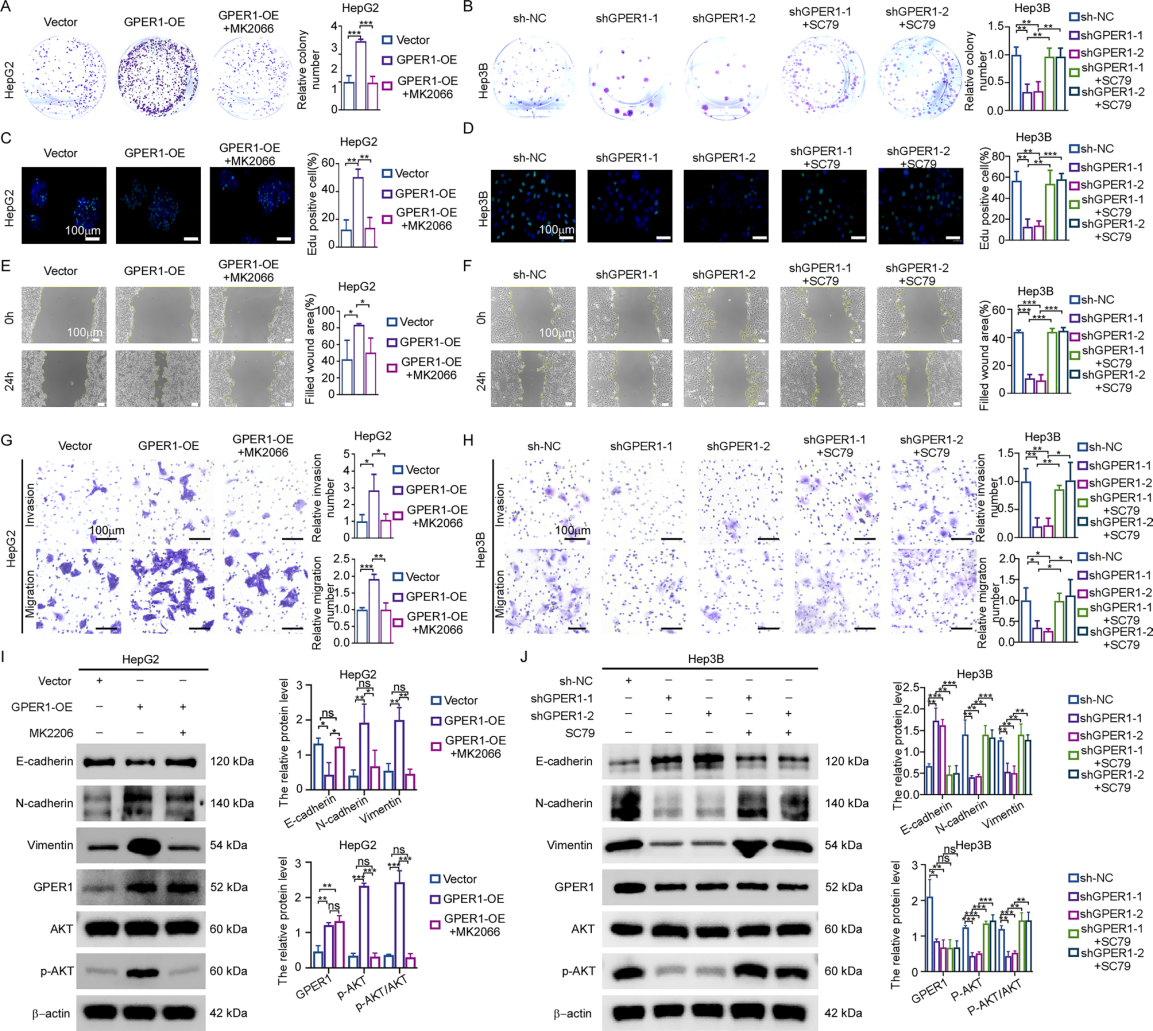
The oncogenic effect of GPER1 in HCC is strictly dependent on AKT signaling activation. (**A**, **B**) Cell proliferation was evaluated using a colony formation assay in GPER1-overexpressing HepG2 cells with MK-2206 treatment (**A**) and in GPER1-knockdown Hep3B cells with SC79 treatment (**B**). (**C**, **D**) EdU assays are performed to assess cell proliferative ability in GPER1-overexpressing HepG2 (**C**) and GPER1-knockdown Hep3B (**D**) cells following altered AKT activity. (**E**, **F**) Wound healing assays verify the effect of GPER1 manipulation combined with AKT inhibition or activation on cell migration ability in HepG2 (**E**) and Hep3B (**F**) cells. (**G**, **H**) Transwell assays are performed to evaluate cell invasion and migration in GPER1-modified HepG2 (**G**) and Hep3B (**H**) cells with pharmacological regulation of AKT signaling. (**I**, **J**) Western blotting of EMT markers and AKT phosphorylation upon GPER1 overexpression with MK-2206 intervention (**I**) and upon GPER1 knockdown with SC79 supplementation (**J**) in HCC cells. Molecular weight markers and quantitative analysis of protein expression levels from three independent experiments are presented on the right. Parametric or non-parametric tests are used as appropriate based on data distribution. Scale bar: 100 μm. Data are presented as mean ± SD; **ns, not significant; *p < 0.05, **p < 0.01, ***p < 0.001

In GPER1-overexpressing HepG2 cells, GPER1 overexpression significantly enhanced cell proliferation, migration, and invasion, as validated by colony formation (Fig. 5A), EdU (Fig. 5C), wound healing (Fig. 5E), and Transwell assays (Fig. 5G). Western blotting analysis further revealed that GPER1 overexpression promoted epithelial-mesenchymal transition (EMT) and increased AKT phosphorylation (Fig. 5I). Notably, co-treatment with MK-2206 (a selective AKT inhibitor) abrogated all these oncogenic effects induced by GPER1 overexpression (Fig. 5A, C, E, G, and I). Conversely, GPER1 knockdown in Hep3B cells suppressed cell proliferation, migration, and invasion (Fig. 5B, D, F, and H), accompanied by inhibited EMT and reduced AKT phosphorylation (Fig. 5J). Supplementation with SC79 (a specific AKT activator) effectively rescued these impaired malignant phenotypes and reversed the molecular changes caused by GPER1 knockdown (Fig. 5B, D, F, H, and J). These results collectively demonstrate that GPER1 functions as an oncogenic regulator in HCC by promoting cell proliferation, migration, invasion, and EMT, and this pro-tumorigenic effect is strictly dependent on AKT signaling activation.

#### GPER1 antagonist G15 and agonist G1 validate the CYP19A1-GPER1-AKT axis in regulating HCC malignant phenotypes

**Fig. 6:**
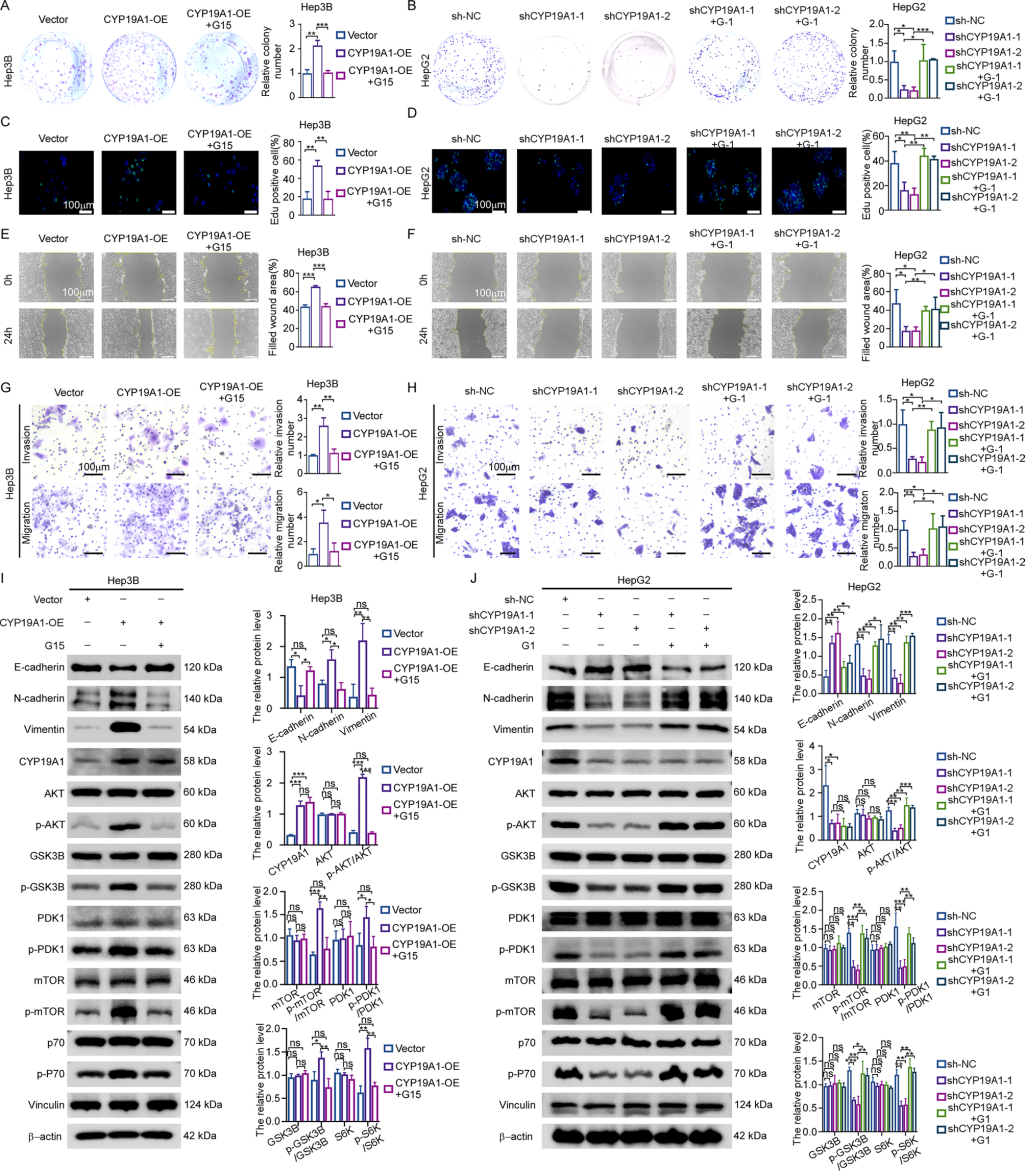
Pharmacological validation of the CYP19A1-GPER1-AKT axis using a GPER1 agonist and antagonist in HCC. (**A**, **B**) Evaluation of cell proliferation using colony formation assays in CYP19A1-overexpressing Hep3B cells treated with G15 (**A**) and in CYP19A1-knockdown HepG2 cells treated with G1 (**B**). (**C**, **D**) Assessment of cell proliferative ability using EdU assays in CYP19A1-overexpressing Hep3B cells (**C**) and CYP19A1-knockdown HepG2 cells (**D**), following GPER1 antagonism or agonism. (**E**, **F**) Wound healing assays assessing the effect of CYP19A1 manipulation on cell migration in Hep3B cells treated with G15 (**E**) and HepG2 cells treated with G1 (**F**). (**G**, **H**) Evaluation of cell invasion and migration using transwell assays in CYP19A1-overexpressing Hep3B (**G**) and CYP19A1-knockdown HepG2 (**H**) cells with GPER1 pharmacological regulation. (**I**, **J**) Western blotting of EMT markers and phosphorylation of AKT and its upstream/downstream regulators in CYP19A1-overexpressing Hep3B cells with G15 co-treatment (**I**) and CYP19A1-knockdown HepG2 cells with G1 supplementation (**J**). Demonstration of molecular weight markers and quantitative analysis of protein expression levels from three independent experiments on the right. Scale bar: 100 μm. Data are presented as mean ± SD; **ns, not significant; *p < 0.05, **p < 0.01, ***p < 0.001

To further confirm that CYP19A1 exerts its oncogenic effects through GPER1-mediated signaling, rescue experiments were performed using GPER1-specific antagonist G15 and agonist G1. In CYP19A1-overexpressing Hep3B cells, treatment with G15 (a GPER1 antagonist) significantly abrogated the enhanced proliferation induced by CYP19A1 overexpression, as evidenced by colony formation (Fig. 6A) and EdU assays (Fig. 6C). Consistently, G15 treatment reversed the promoting effects of CYP19A1 overexpression on Hep3B cell migration and invasion, as confirmed by wound healing (Fig. 6E) and Transwell assays (Fig. 6G). Western blotting analysis revealed that CYP19A1 overexpression in Hep3B cells upregulated EMT markers (N-cadherin and vimentin), downregulated E-cadherin, and increased the phosphorylation levels of AKT and its upstream/downstream regulators (PDK1, GSK3β, p-S6K, and mTORC1). All these effects were significantly reversed using G15 co-treatment (Fig. 6I). Conversely, in CYP19A1-knockdown HepG2 cells, supplementation with G1 (a GPER1-specific agonist) restored the suppressed cell proliferation, as confirmed using colony formation (Fig. 6B) and EdU assays (Fig. 6D). Additionally, G1 treatment rescued the impaired migration and invasion capabilities of CYP19A1-knockdown HepG2 cells, as demonstrated by wound healing (Fig. 6F) and Transwell assays (Fig. 6H). Mechanistically, Western blotting analysis indicated that CYP19A1 knockdown in HepG2 cells inhibited EMT and reduced phosphorylation of key components of the AKT pathway, including AKT, PDK1, GSK3β, p-S6K, and mTORC1. These inhibitory effects were efficiently rescued by G1 supplementation (Fig. 6J). Collectively, these findings indicate that CYP19A1 drives malignant phenotypes and AKT pathway activation in HCC in a GPER1-dependent manner, with GPER1 antagonism reversing and agonism rescuing the effects of CYP19A1 modulation.

### Reciprocal genetic validation of the CYP19A1-GPER1-AKT signaling cascade in HCC

**Fig. 7:**
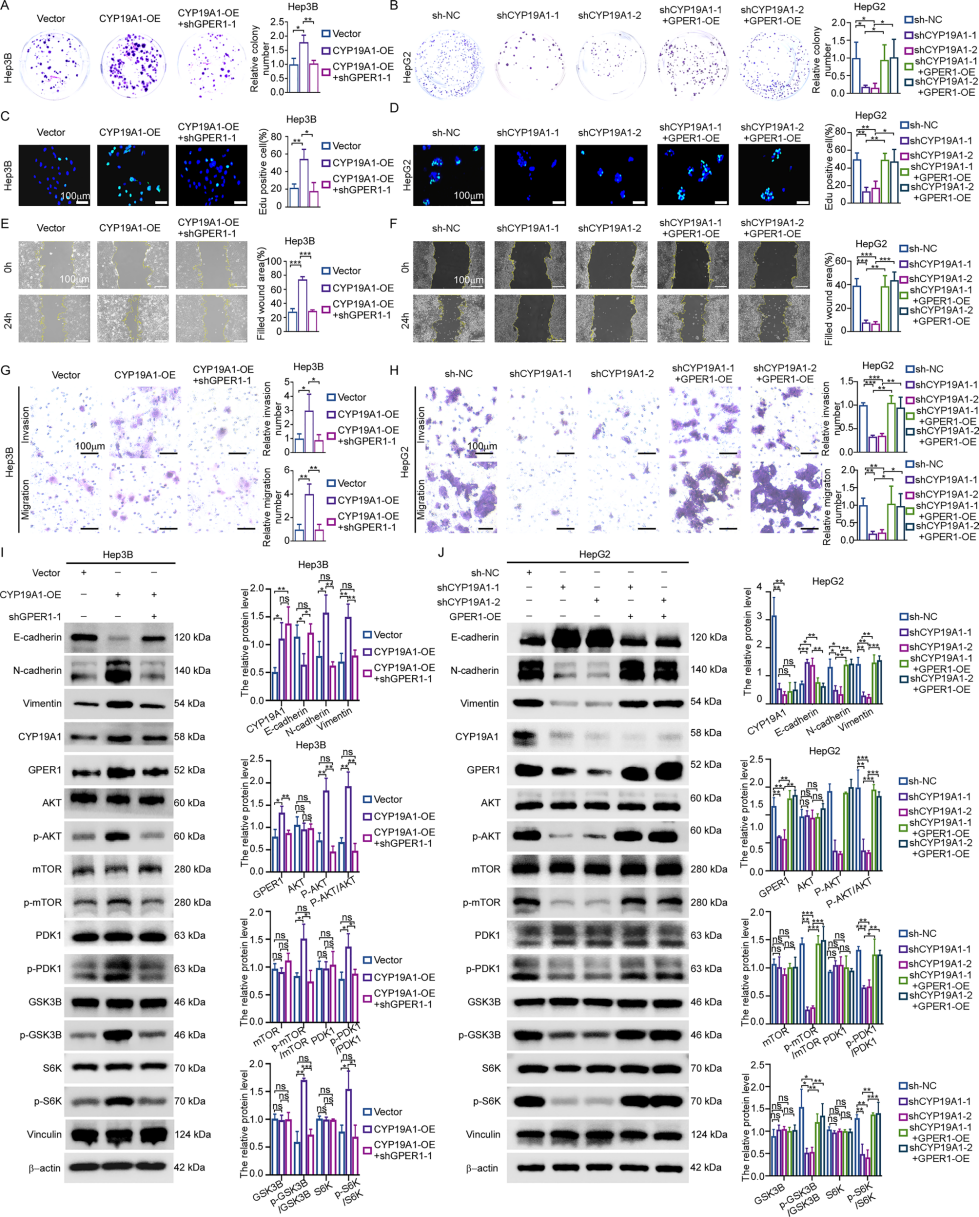
Genetic validation of the CYP19A1-GPER1-AKT axis through reciprocal endogenous rescue in HCC. (**A**, **B**) Colony formation assays verify that GPER1 reverses the influence of CYP19A1 on cell proliferation in Hep3B (**A**) and HepG2 (**B**) cells. (**C**, **D**) EdU assays verify that GPER1 reverses the effect of CYP19A1 on cell proliferation in Hep3B (**C**) and HepG2 (**D**) cells. (**E**, **F**) Wound healing assays verify that GPER1 reverses the effect of CYP19A1 on cell proliferation in Hep3B (**E**) and HepG2 (**F**) cells. (**G**, **H**) Transwell assays verify that GPER1 reverses the effect of CYP19A1 on cell migration and invasion in Hep3B (**G**) and HepG2 (**H**) cells. (**I**, **J**) Western blotting of EMT markers and AKT phosphorylation in Hep3B (**I**) and HepG2 (**J**) cells. Molecular weight markers and quantitative analysis of protein expression levels from three independent experiments are presented on the right. Parametric or non-parametric tests are used as appropriate based on data distribution. Scale bar: 100 μm. Data are presented as mean ± SD; **ns, not significant; *p < 0.05, **p < 0.01, ***p < 0.001

To genetically confirm whether GPER1 is required for CYP19A1-driven malignant phenotypes in HCC, reciprocal genetic rescue experiments were performed. GPER1 was knocked down in CYP19A1-overexpressing Hep3B cells, whereas GPER1 was overexpressed in CYP19A1-silenced HepG2 cells. Functional assays revealed GPER1 as a dominant regulator downstream of CYP19A1. Colony formation and EdU assays demonstrated that the enhanced proliferation induced by CYP19A1 overexpression in Hep3B cells was markedly attenuated following GPER1 depletion. Conversely, the suppressed proliferation observed in CYP19A1-knockdown HepG2 cells was effectively restored upon GPER1 overexpression (Fig. 7A–D). Wound-healing assays illustrated that GPER1 inhibition reversed the pro-migratory effect caused by elevated CYP19A1 in Hep3B cells, while GPER1 supplementation rescued impaired migration in CYP19A1-deficient HepG2 cells (Fig. 7E and F). Transwell assays confirmed that GPER1 knockdown abrogated CYP19A1-facilitated migration and invasion in Hep3B cells, whereas GPER1 overexpression recovered these compromised invasive capacities in HepG2 cells (Fig. 7G and H). Mechanistically, Western blotting analysis was performed to detect alterations in EMT markers and key components of the AKT signaling pathway (Fig. 7I and J). In Hep3B cells, CYP19A1 overexpression upregulated the mesenchymal markers N-cadherin and vimentin, downregulated the epithelial marker E-cadherin, and increased the phosphorylation levels of AKT and its upstream and downstream regulators, including PDK1, GSK3β, p-S6K, and mTORC1. Notably, all of these molecular alterations triggered by CYP19A1 were significantly reversed following subsequent GPER1 knockdown. In contrast, CYP19A1 depletion in HepG2 cells inhibited EMT progression and reduced the phosphorylation of AKT, PDK1, GSK3β, p-S6K, and mTORC1. Importantly, GPER1 overexpression effectively reversed the inhibitory effects of CYP19A1 silencing. Collectively, these rescue experiments identify the GPER1 as a critical downstream effector of CYP19A1, promoting proliferation, migration, invasion, EMT activation, and constitutive AKT cascade activation in HCC.

### In vivo validation of the CYP19A1-GPER1 oncogenic axis in HCC

**Fig. 8:**
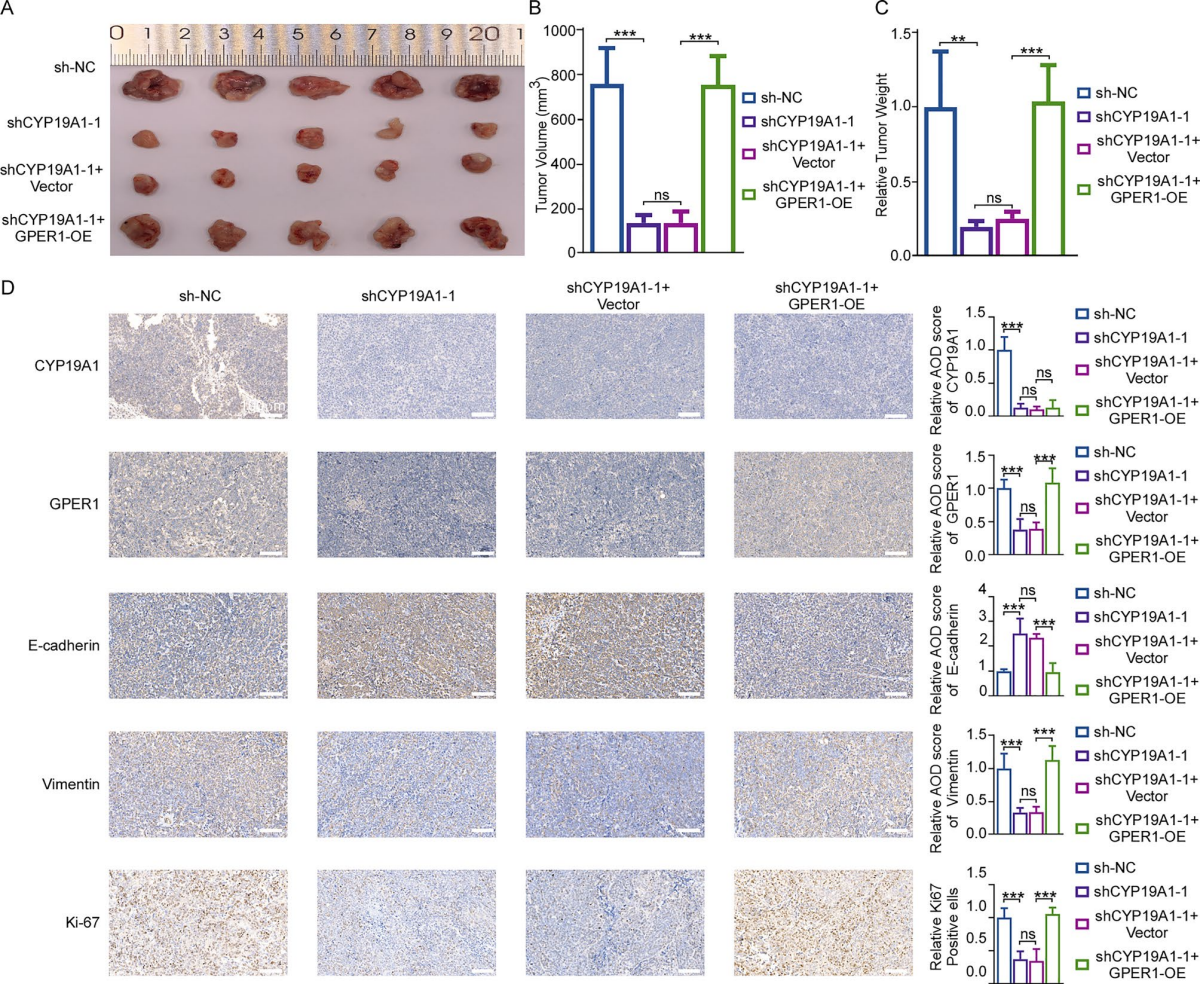
In vivo validation of the CYP19A1-GPER1 axis in subcutaneous xenograft models. (**A**) Subcutaneous tumors representative image. (**B**, **C**) Quantification of tumor volume (**B**) and relative tumor weight (**C**). (**D**) IHC staining of subcutaneous tumors for CYP19A1, GPER1, vimentin, E-cadherin, N-cadherin, and Ki-67. Quantitative analysis of the relative AOD score for each protein and the percentage of Ki-67-positive cells is presented on the right. HepG2 cells transduced with sh-NC (control shRNA), shCYP19A1-1 (CYP19A1 knockdown), shCYP19A1-1 + vector (CYP19A1 knockdown with empty vector control), or shCYP19A1-1 + GPER1-OE (CYP19A1 knockdown with GPER1 overexpression) are injected orthotopically into the livers of nude mice. Parametric or non-parametric tests are used as appropriate based on data distribution. Scale bar: 100 μm. Data are presented as mean ± SD; **ns, not significant; *p < 0.05, **p < 0.01, ***p < 0.001

**Fig. 9:**
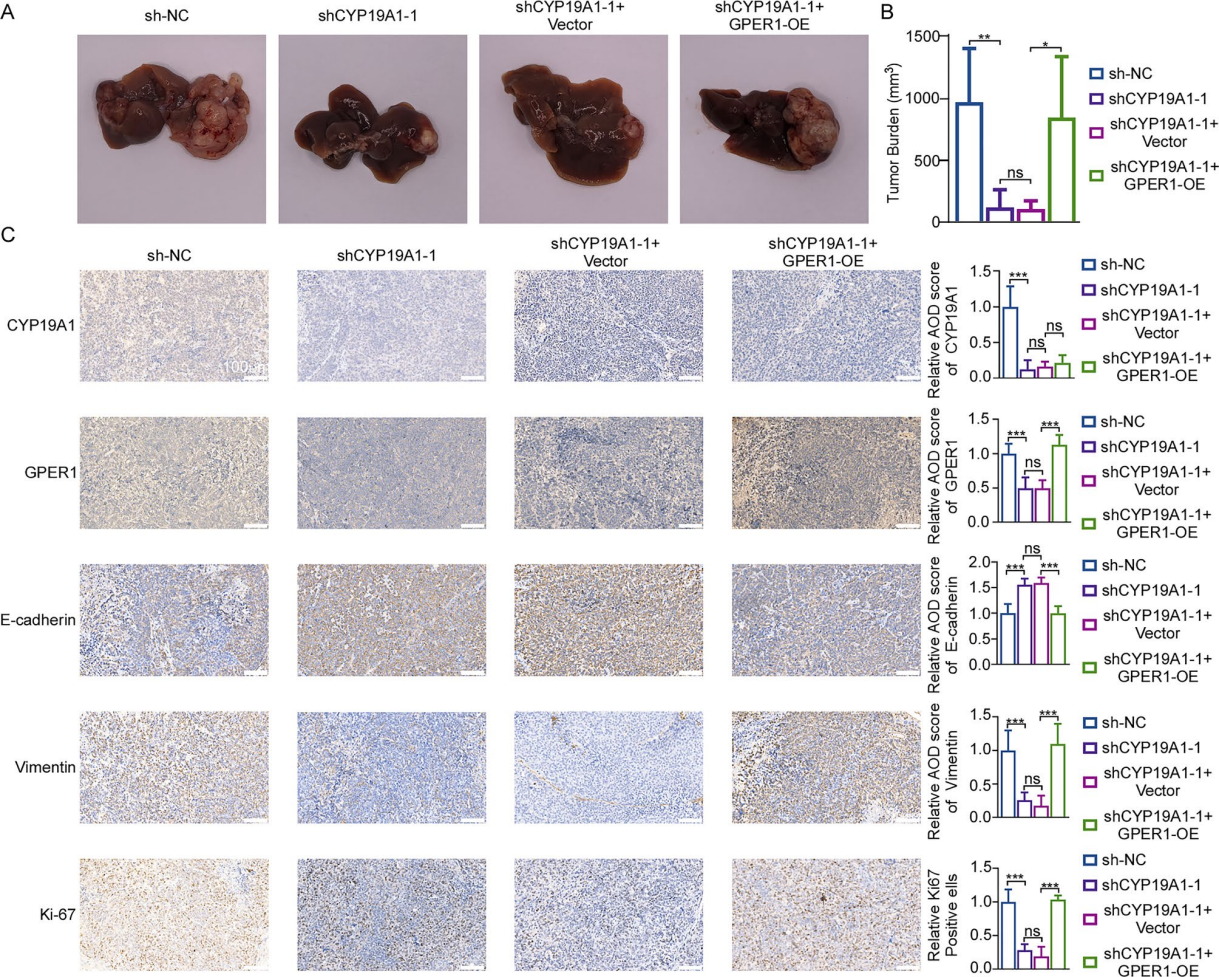
In vivo validation of the CYP19A1-GPER1 axis in orthotopic xenograft models. (**A**) Representative images of liver tumors. (**B**) Quantification of tumor burden. (**C**) IHC staining of orthotopic liver tumors for CYP19A1, vimentin, E-cadherin, N-cadherin, GPER1, and Ki-67. Quantitative analysis of the relative AOD score for each protein and the percentage of Ki-67-positive cells is presented on the right. HepG2 cells transduced with sh-NC (control shRNA), shCYP19A1-1 (CYP19A1 knockdown), shCYP19A1-1 + vector (CYP19A1 knockdown with empty vector control), or shCYP19A1-1 + GPER1-OE (CYP19A1 knockdown with GPER1 overexpression) are injected subcutaneously into nude mice. Parametric or non-parametric tests are used as appropriate based on data distribution. Scale bar: 100 μm. Data are presented as mean ± SD; **ns, not significant; *p < 0.05, **p < 0.01, ***p < 0.001

In vivo validation using stably modified HepG2 xenografts demonstrated that CYP19A1 knockdown significantly reduced tumor growth in subcutaneous (decreased tumor volume and mass, Fig. 8A–C) and orthotopic (diminished hepatic tumor burden, Fig. 9A and B) mouse models, with GPER1 overexpression rescuing this suppression. Immunohistochemical analysis of subcutaneous tumors revealed that CYP19A1 knockdown decreased Ki-67, N-cadherin, and vimentin expression while increasing E-cadherin levels-alterations reversed by GPER1 co-overexpression (Fig. 8D). Consistently, orthotopic tumors displayed identical molecular changes: reduced Ki-67, N-cadherin and vimentin alongside elevated E-cadherin, with GPER1 overexpression similarly reversing these marker alterations (Fig. 9C), mechanistically confirming GPER1 maintains CYP19A1-driven tumor progression. These in vivo findings highlight GPER1 as a therapeutically targetable node essential for sustaining CYP19A1-driven HCC progression.

## Discussion

In this study, we analyzed genes involved in lipid metabolism in HCC and identified CYP19A1 as a pivotal prognostic hub. Its oncogenic effects are mediated through a functional interaction with GPER1. Our integrated analysis of gene expression, clinical data, and functional experiments identified the CYP19A1-GPER1 axis as a critical node linking lipid metabolic dysregulation, immune modulation, and aggressive forms of HCC. This axis may be significant for predicting patient outcomes and developing targeted therapies.

Our initial bioinformatic analysis of TCGA-LIHC data revealed distinct lipid metabolic signatures in HCC tissues. These included enrichment in pathways governing fatty acid metabolism, peroxisome function, and PPAR signaling. This is consistent with prior observations that perturbed lipid homeostasis fuels HCC growth and metastasis. Within this landscape, CYP19A1 emerged as a central hub through its dual presence in the PPI network and association with survival outcomes, distinguishing it from other CYP family members (CYP2C9 and CYP7A1) identified in our intersection analysis. CYP7A1 promotes bile acid synthesis, participates in lipid metabolism disorders, and is involved in inflammation-cancer transformation processes, thereby contributing to HCC carcinogenesis and progression [41–44]. CYP2C9 reduces the cytotoxicity of chemotherapeutic drugs by participating in drug metabolism, thereby promoting chemoresistance in HCC [44]. This prominence aligns with CYP19A1‘s established role as a metabolic gatekeeper in estrogen biosynthesis, though its role in HCC remains poorly defined.

Our finding that CYP19A1 is overexpressed in HCC and correlates with advanced disease stages (T and BCLC) and reduced survival, reinforces its clinical importance. This overexpression was validated across multiple platforms—from TCGA transcriptomics to patient-derived qPCR, IHC, and Western blotting—confirming CYP19A1 as an independent prognostic biomarker. Notably, GSEA revealed that CYP19A1 expression negatively correlates with fatty acid metabolism and peroxisomal pathways, while positively associating with pro-tumorigenic signaling (PI3K-Akt and JAK-STAT) and extracellular matrix degradation. This dichotomy suggests CYP19A1 may drive HCC progression by rewiring lipid metabolic flux away from homeostatic processes toward pathways supporting proliferation and invasion, as supported by our functional assays demonstrating enhanced proliferation, migration, and invasion in CYP19A1-overexpressing cells. As a key enzyme catalyzing estrogen biosynthesis, CYP19A1 produces estrogens that may serve as ligands for GPER1, thereby activating downstream signaling pathways. In colon cancer, estrogens derived from CYP19A1 may also affect HCC microenvironment via GPER1: CYP19A1-induced estrogens upregulate PD-L1, IL-6, and TGF-β through the GPER1 signaling pathway, thereby suppressing CD8^+^ T cell function. In summary, we hypothesize that CYP19A1 may promote HCC progression by activating GPER1.

A key novel finding is that GPER1 functions as a critical effector of CYP19A1-mediated oncogenesis. Rescue experiments in Hep3B and HepG2 cells revealed that GPER1 depletion abrogates CYP19A1-induced proliferation, migration, invasion, and EMT. However, these phenotypes are rescued by GPER1 overexpression following CYP19A1 knockdown. Mechanistically, we linked this axis to AKT activation—a central node in HCC progression—and EMT marker dysregulation (E-cadherin downregulation and N-cadherin/vimentin upregulation), providing a molecular framework for their cooperative effects. This finding expands prior evidence of GPER1-mediated regulation of macrophage function and immune evasion in HCC, positioning the CYP19A1-GPER1 axis at the intersection of metabolic dysregulation and immune modulation.

The in vivo validation in subcutaneous and orthotopic xenograft models further strengthens this axis’s biological significance. CYP19A1 knockdown reduced tumor burden and altered proliferation and EMT markers, with GPER1 overexpression reversing these effects—confirming that GPER1 is not merely associated with, but functionally required for CYP19A1-driven tumor growth.

These preclinical data support the therapeutic potential of targeting this axis. To further explore the functional relevance of CYP19A1 in drug response, we performed in vitro assays. Functional assays further demonstrated that CYP19A1 overexpression increased HCC cell viability under sorafenib treatment, while CYP19A1 knockdown made cells more sensitive to sorafenib, suggesting that CYP19A1 expression may influence response to this therapeutic agent. Molecular docking simulations also indicated potential interactions between CYP19A1 and sorafenib, lenvatinib, and regorafenib; however, functional validation for lenvatinib and regorafenib remains absent. These docking-based findings should be viewed as preliminary and require further experimental investigation.

Our study presents novelty in several aspects. First, while CYP19A1 has been implicated in estrogen biosynthesis and other cancers, its functional role in HCC and its mechanistic link to GPER1 have not been previously established. Our study is the first to demonstrate that CYP19A1 drives HCC progression specifically through GPER1-dependent AKT activation. Second, we provide genetic and pharmacological evidence that GPER1 is an indispensable downstream mediator of CYP19A1, using reciprocal rescue experiments and GPER1-specific agonist/antagonist interventions. Third, beyond establishing this axis, we further validate its translational potential by demonstrating that CYP19A1 expression modulates sorafenib sensitivity in HCC cells.

Comparison with previous studies provides additional context for these findings. Although GPER1 promotes HCC progression through activation of PI3K-AKT-mTORC1 signaling [34], its upstream regulatory mechanism remains unclear. Our study identifies CYP19A1 as an essential upstream regulator of GPER1, providing a missing link between estrogen biosynthesis and GPER1 activation. Moreover, although CYP19A1 has been examined in gastric and colorectal cancers [45, 46], its functional role in HCC and its specific interaction with GPER1 have not been previously reported. Accordingly, our findings expand the functional landscape of CYP19A1 to include HCC and reveal a previously unrecognized oncogenic axis. Collectively, these insights advance the field by linking estrogen signaling to AKT activation in HCC, providing new opportunities for prognostic stratification and targeted therapy.

Several limitations of our study should be acknowledged. First, although TCGA-LIHC and patient validation offer strong clinical correlation, larger multi-center cohorts are necessary to confirm CYP19A1‘s prognostic value, especially across diverse ethnic groups and in different etiological contexts. Second, the mechanistic link between CYP19A1, estrogen biosynthesis, and GPER1 activation needs further investigation—specifically, whether CYP19A1-derived estrogens directly activate GPER1 or if other ligands or non-canonical pathways are involved. Third, our in vivo models utilize immunocompromised mice, which limits understanding of how this axis influences the adaptive immune response, critical for HCC immunotherapy success. Fourth, while GSEA analysis suggested an association between CYP19A1 and lipid metabolism pathways, we did not experimentally validate this connection, since our primary focus was on establishing the CYP19A1-GPER1-AKT signaling pathway. The potential role of lipid metabolism in this pathway remains an intriguing observation from bioinformatics and merits further study. Fifth, although our functional data support that CYP19A1-derived estrogens activate GPER1, we did not conduct direct experiments such as competitive binding assays or measurements of estrogen-GPER1 interactions. Future research should employ techniques like surface plasmon resonance or fluorescence polarization to confirm this interaction directly.

Future directions should focus on translating these findings into clinical applications. Therapeutically, combining CYP19A1 inhibitors with GPER1 antagonists could disrupt this axis, potentially synergizing with existing therapies like anti-angiogenics or checkpoint inhibitors. Additionally, given the functional link between CYP19A1 and sorafenib sensitivity, further investigation of CYP19A1 as a predictive biomarker for sorafenib response is warranted. The role of CYP19A1 in modulating sensitivity to lenvatinib or regorafenib remains unclear. Mechanistic studies of the CYP19A1-GPER1 axis with metabolic pathways and immune checkpoints may identify combinatorial targeting opportunities.

In conclusion, our study identifies the CYP19A1-GPER1 axis as a novel, therapeutically actionable node in HCC, connecting lipid metabolic dysregulation, pro-tumorigenic signaling, and immune modulation. These findings advance our understanding of HCC pathogenesis and provide a rationale for developing targeted strategies to disrupt this axis, ultimately improving outcomes for patients with this aggressive malignancy.

## Conclusions

In summary, our study identifies the CYP19A1-GPER1 axis as a critical oncogenic driver in HCC. Clinically, CYP19A1 overexpression correlates with advanced tumor stages and poor survival, establishing it as a promising prognostic biomarker. Mechanistically, we demonstrate that GPER1 is indispensable for CYP19A1-driven AKT activation, EMT, and malignant progression. These findings establish the CYP19A1-GPER1 axis as a potential therapeutic target. Prospective studies using CYP19A1 inhibitors or GPER1 antagonists in combination with standard therapies (for example, sorafenib) are warranted to evaluate clinical translation. Ultimately, targeting this axis may offer a novel strategy to improve outcomes for HCC patients.

## Electronic supplementary material

Below is the link to the electronic supplementary material.

Supplementary Material 1

Supplementary Material 2

Supplementary Material 3

## Data Availability

All data generated or analyzed during this study are included in this published article and its supplementary files; public datasets are available in The Cancer Genome Atlas (TCGA-LIHC).
