# Supplementary material for: Identification of the CYP19A1-GPER1 axis as a critical oncogenic driver in hepatocellular carcinoma via AKT activation
Source: J Transl Med. 2026 May 19;24:896. doi: 10.1186/s12967-026-08246-3 (PMC13366950; doi:10.1186/s12967-026-08246-3)
Supplement: Supplementary file 1 — Supplementary Material 1 [file 12967_2026_8246_MOESM1_ESM.docx]

**Table S1.** The primers (5’-3’) used in this study.

| Primers for RT-qPCR | | | | 5’-3’ |
| --- | --- | --- | --- | --- |
|  | CYP19A1 | Forward | ATCGGATCCCTGCCTGTGAC | |
|  |  | Reverse | TGGCTGATGCTCTGCTGAGG | |
|  | β-actin | Forward | GCCGGACTCATCGTACTCC | |
|  |  | Reverse | GTGACGTTGACATCCGTAAAGA | |

**Table S2.** Effective sequences (5’-3’) of lentivirus plasmids-overexpression.

| Plasmids | 5’-3’ |
| --- | --- |
| LV- CYP19A1  LV-GPER1 | atggttttggaaatgctgaacccgatacattataacatcaccagcatcgtgcctgaagccatgcctgctgccaccatgccagtcctgctcctcactggcctttttctcttggtgtggaattatgagggcacatcctcaataccaggtcctggctactgcatgggaattggacccctcatctcccacggcagattcctgtggatggggatcggcagtgcctgcaactactacaaccgggtatatggagaattcatgcgagtctggatctctggagaggaaacactcattatcagcaagtcctcaagtatgttccacataatgaagcacaatcattacagctctcgattcggcagcaaacttgggctgcagtgcatcggtatgcatgagaaaggcatcatatttaacaacaatccagagctctggaaaacaactcgacccttctttatgaaagctctgtcaggccccggccttgttcgtatggtcacagtctgtgctgaatccctcaaaacacatctggacaggttggaggaggtgaccaatgaatcgggctatgtggacgtgttgacccttctgcgtcgtgtcatgctggacacctctaacacgctcttcttgaggatccctttggacgaaagtgctatcgtggttaaaatccaaggttattttgatgcatggcaagctctcctcatcaaaccagacatcttctttaagatttcttggctatacaaaaagtatgagaagtctgtcaaggatttgaaagatgccatagaagttctgatagcagaaaaaagacgcaggatttccacagaagagaaactggaagaatgtatggactttgccactgagttgattttagcagagaaacgtggtgacctgacaagagagaatgtgaaccagtgcatattggaaatgctgatcgcagctcctgacaccatgtctgtctctttgttcttcatgctatttctcattgcaaagcaccctaatgttgaagaggcaataataaaggaaatccagactgttattggtgagagagacataaagattgatgatatacaaaaattaaaagtgatggaaaacttcatttatgagagcatgcggtaccagcctgtcgtggacttggtcatgcgcaaagccttagaagatgatgtaatcgatggctacccagtgaaaaaggggacaaacattatcctgaatattggaaggatgcacagactcgagtttttccccaaacccaatgaatttactcttgaaaattttgcaaagaatgttccttataggtactttcagccatttggctttgggccccgtggctgtgcaggaaagtacatcgccatggtgatgatgaaagccatcctcgttacacttctgagacgattccacgtgaagacattgcaaggacagtgtgttgagagcatacagaagatacacgacttgtccttgcacccagatgagactaaaaacatgctggaaatgatctttaccccaagaaactcagacaggtgtctggaacac  ATGGATGTGACTTCCCAAGCCCGGGGCGTGGGCCTGGAGATGTACCCAGGCACCGCGCAGCCTGCGGCCCCCAACACCACCTCCCCCGAGCTCAACCTGTCCCACCCGCTCCTGGGCACCGCCCTGGCCAATGGGACAGGTGAGCTCTCGGAGCACCAGCAGTACGTGATCGGCCTGTTCCTCTCGTGCCTCTACACCATCTTCCTCTTCCCCATCGGCTTTGTGGGCAACATCCTGATCCTGGTGGTGAACATCAGCTTCCGCGAGAAGATGACCATCCCCGACCTGTACTTCATCAACCTGGCGGTGGCGGACCTCATCCTGGTGGCCGACTCCCTCATTGAGGTGTTCAACCTGCACGAGCGGTACTACGACATCGCCGTCCTGTGCACCTTCATGTCGCTCTTCCTGCAGGTCAACATGTACAGCAGCGTCTTCTTCCTCACCTGGATGAGCTTCGACCGCTACATCGCCCTGGCCAGGGCCATGCGCTGCAGCCTGTTCCGCACCAAGCACCACGCCCGGCTGAGCTGTGGCCTCATCTGGATGGCATCCGTGTCAGCCACGCTGGTGCCCTTCACCGCCGTGCACCTGCAGCACACCGACGAGGCCTGCTTCTGTTTCGCGGATGTCCGGGAGGTGCAGTGGCTCGAGGTCACGCTGGGCTTCATCGTGCCCTTCGCCATCATCGGCCTGTGCTACTCCCTCATTGTCCGGGTGCTGGTCAGGGCGCACCGGCACCGTGGGCTGCGGCCCCGGCGGCAGAAGGCGCTCCGCATGATCCTCGCGGTGGTGCTGGTCTTCTTCGTCTGCTGGCTGCCGGAGAACGTCTTCATCAGCGTGCACCTCCTGCAGCGGACGCAGCCTGGGGCCGCTCCCTGCAAGCAGTCTTTCCGCCATGCCCACCCCCTCACGGGCCACATTGTCAACCTCGCCGCCTTCTCCAACAGCTGCCTAAACCCCCTCATCTACAGCTTTCTCGGGGAGACCTTCAGGGACAAGCTGAGGCTGTACATTGAGCAGAAAACAAATTTGCCGGCCCTGAACCGCTTCTGTCACGCTGCCCTGAAGGCCGTCATTCCAGACAGCACCGAGCAGTCGGATGTGAGGTTCAGCAGTGCCGTG |

**Table S3.** Effective sequences (5’-3’) of lentivirus plasmids-knockdown.

| Plasmids | 5’-3’ |
| --- | --- |
| sh-NC | TTCTCCGAACGTGTCACGTCTCGAGACGTGACACGT TCG GAG AATTTTTG |
| shCYP19A1-1 | ACCGGCGTTACACTTCTGAGACGATTCTCGAGAATCGTCTCAGAAGTGTAACGTTTTTT |
| shCYP19A1-2 | CAATCATTACAGCTCUCGAUCTCGAGATCGAGAGCTGTAATGATTGTTTTTG |
| shGPER1-1 | GGATGAGCTTCGACCGCTACTCGAGTAGCGGTCGAAGCTCATCCTTTTTG |
| shGPER1-2 | CCGACCTGTACTTCATCAACTCGAGTTGATGAAGTACAGGTCGGTTTTTG |

**Supplementary Figure 1**


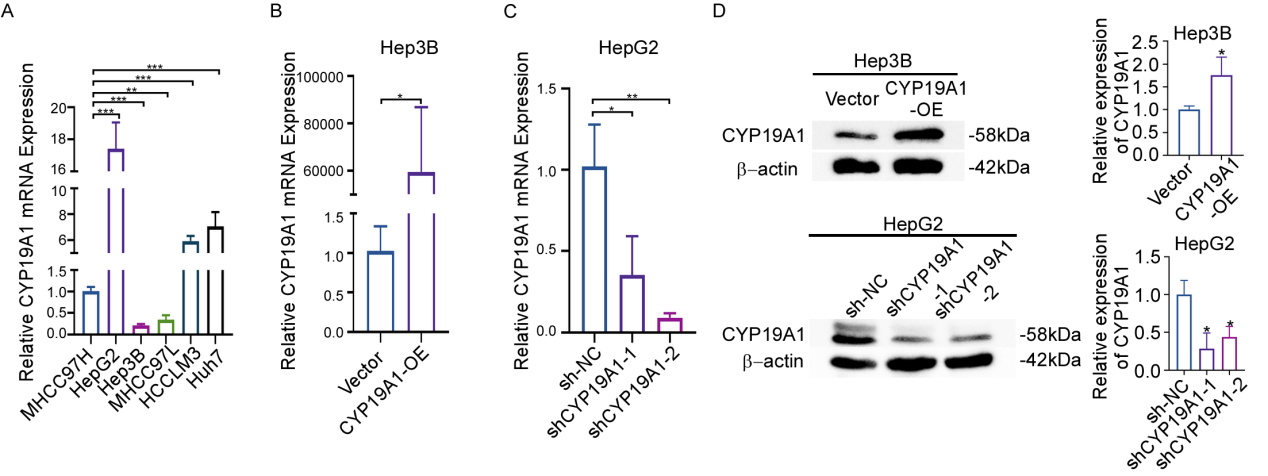


**Supplementary Figure 1**. **CYP19A1 expression profiling and genetic manipulation in HCC cell lines.** (**A)** CYP19A1 mRNA expression in hepatocellular carcinoma cell lines as determined by RT-qPCR. (**B)** Validation of CYP19A1 overexpression in Hep3B cells by RT-qPCR. **(C)** RT-qPCR confirmation of CYP19A1 knockdown efficiency in HepG2 cells **(D)**Western blot validation of CYP19A1 overexpression in Hep3B cells and knockdown in HepG2 cells.Data are denoted as mean ± SD from three independent experiments.*P < 0.05; **P < 0.01; ***P < 0.001.

**Supplementary Figure 2**


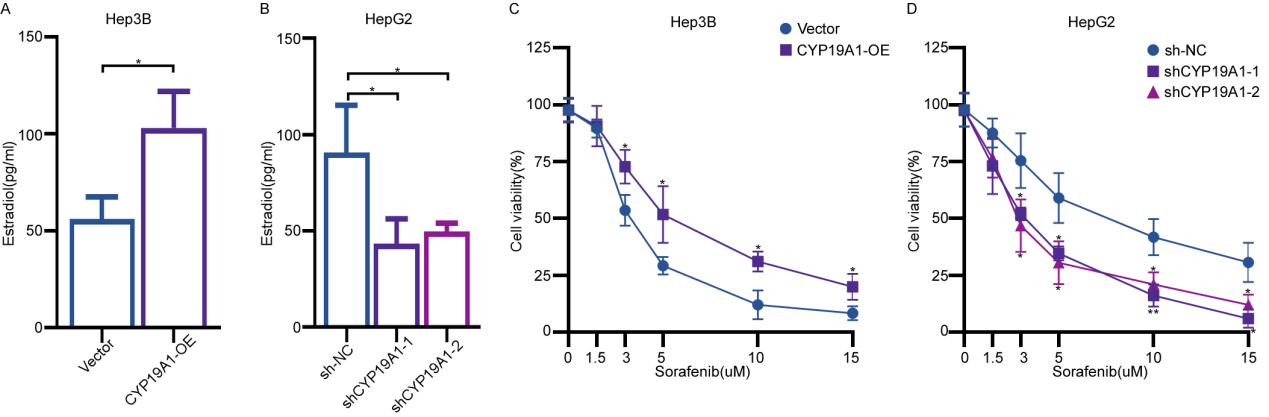


**Supplementary Figure S2. CYP19A1 regulates estrogen production and modulates sorafenib sensitivity in HCC cells. (A, B)** 17β-estradiol (E2) levels in conditioned media of Hep3B cells with CYP19A1 overexpression (A) and HepG2 cells with CYP19A1 knockdown (B) measured by ELISA (mean ± SD; *p < 0.05). **(C, D)** Cell viability of Hep3B cells with CYP19A1 overexpression (C) and HepG2 cells with CYP19A1 knockdown (D) treated with sorafenib, assessed by CCK-8 assay. Data are presented as mean ± SD from three independent experiments. ns, not significant; *p < 0.05, **p < 0.01, ***p < 0.001.
