# Supplementary figures and images for: Identification of the CYP19A1-GPER1 axis as a critical oncogenic driver in hepatocellular carcinoma via AKT activation
Source: J Transl Med. 2026 May 19;24:896. doi: 10.1186/s12967-026-08246-3 (PMC13366950; doi:10.1186/s12967-026-08246-3)

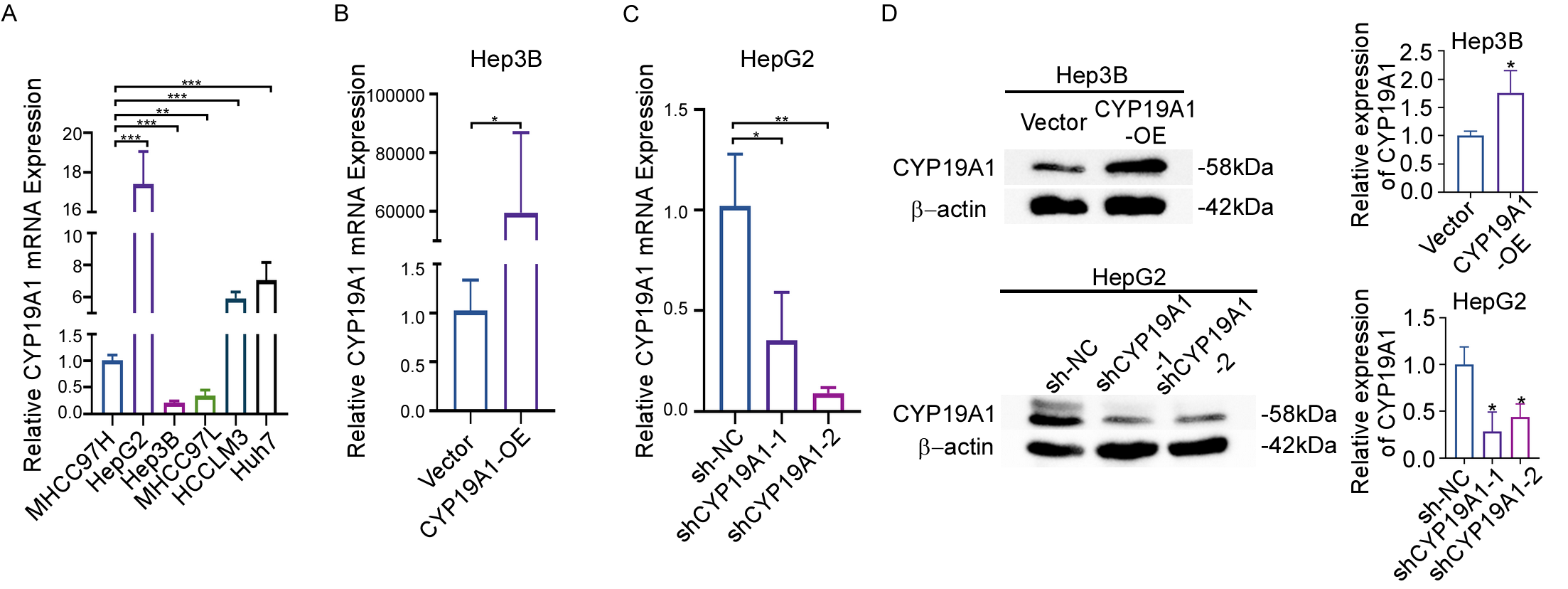

Supplement: Supplementary file 2 — Supplementary Material 2 [file 12967_2026_8246_MOESM2_ESM.tif]

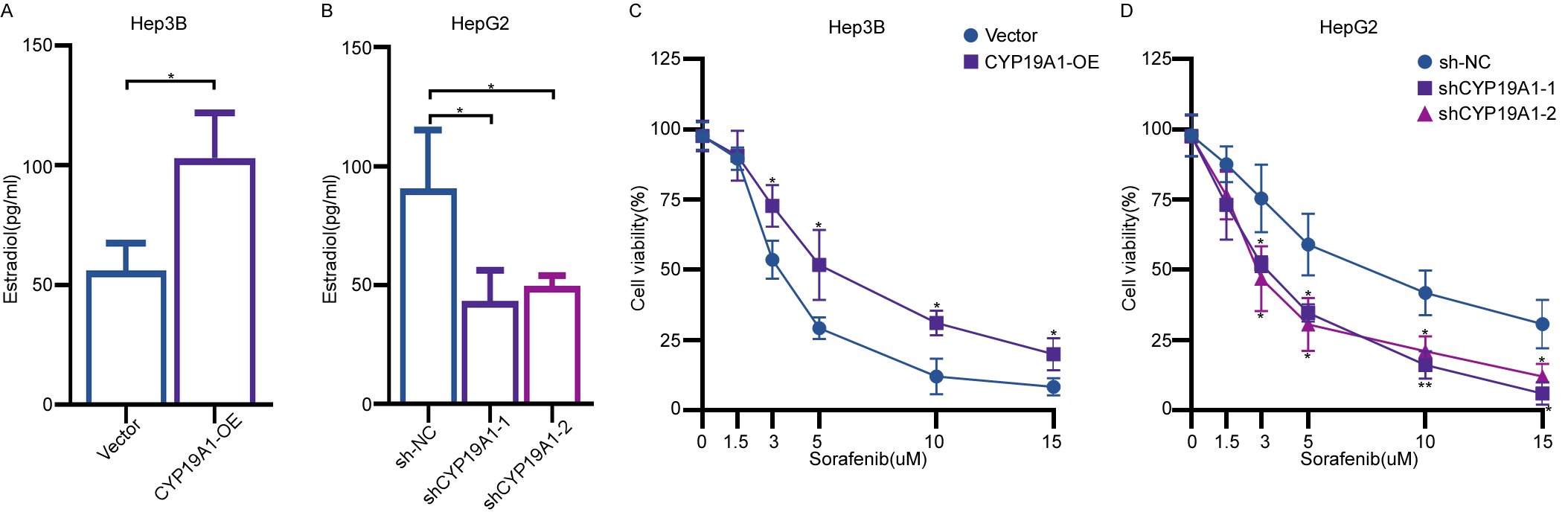

Supplement: Supplementary file 3 — Supplementary Material 3 [file 12967_2026_8246_MOESM3_ESM.tif]
